# Elucidating the Functional Relationship Between Working Memory Capacity and Psychometric Intelligence: A Fixed-Links Modeling Approach for Experimental Repeated-Measures Designs

**DOI:** 10.5709/acp-0166-6

**Published:** 2015-03-31

**Authors:** Philipp Thomas, Thomas Rammsayer, Karl Schweizer, Stefan Troche

**Affiliations:** 1 University of Bern, Department of Psychology and Center for Cognition, Learning and Memory; 2 Johann Wolfgang Goethe University Frankfurt, Department of Psychology; 3 University of Witten / Herdecke, Department of Psychology and Psychotherapy

**Keywords:** working memory capacity, fluid intelligence, fixed-links modeling, confirmatory factor analysis

## Abstract

Numerous studies reported a strong link between working memory capacity (WMC) and fluid intelligence (G*f*), although views differ in respect to how close these two constructs are related to each other. In the present study, we used a WMC task with five levels of task demands to assess the relationship between WMC and G*f* by means of a new methodological approach referred to as fixed-links modeling. Fixed-links models belong to the family of confirmatory factor analysis (CFA) and are of particular interest for experimental, repeated-measures designs. With this technique, processes systematically varying across task conditions can be disentangled from processes unaffected by the experimental manipulation. Proceeding from the assumption that experimental manipulation in a WMC task leads to increasing demands on WMC, the processes systematically varying across task conditions can be assumed to be WMC-specific. Processes not varying across task conditions, on the other hand, are probably independent of WMC. Fixed-links models allow for representing these two kinds of processes by two independent latent variables. In contrast to traditional CFA where a common latent variable is derived from the different task conditions, fixed-links models facilitate a more precise or purified representation of the WMC-related processes of interest. By using fixed-links modeling to analyze data of 200 participants, we identified a non-experimental latent variable, representing processes that remained constant irrespective of the WMC task conditions, and an experimental latent variable which reflected processes that varied as a function of experimental manipulation. This latter variable represents the increasing demands on WMC and, hence, was considered a purified measure of WMC controlled for the constant processes. Fixed-links modeling showed that both the purified measure of WMC (β = .48) as well as the constant processes involved in the task (β = .45) were related to G*f*. Taken together, these two latent variables explained the same portion of variance of G*f* as a single latent variable obtained by traditional CFA (β = .65) indicating that traditional CFA causes an overestimation of the effective relationship between WMC and G*f*. Thus, fixed-links modeling provides a feasible method for a more valid investigation of the functional relationship between specific constructs.

## Introduction

Since Galton’s (1869) first attempt to show that individuals differ in their mental capacities, the area of intelligence has been one of the most fascinating ones in psychology. Besides questions about the structure of intelligence, a lot of research focused on the question of why people perform differently in intelligence tests. A still increasing number of explanations have been investigated, such as brain volume, amplitude and latency of event-related brain potentials, cerebral glucose consumption, and nerve conduction velocity (Vernon, Wickett, Bazana, & Stelmack, 2000). The most widely examined cognitive correlates of psychometric intelligence are speed of information processing (Deary, 2000), attention (Hunt & Lansman, 1982; Schweizer, Moosbrugger, & Goldhammer, 2005), and working memory capacity (WMC; Ackerman, Beier, & Boyle, 2005), with the latter one being of particular interest in the last 20 years.

WMC can be defined as the ability to store and process information simultaneously (Daneman & Carpenter, 1980; for a review see Miyake & Shah, 1999), or as the ability to build and maintain arbitrary bindings of information (Oberauer, Süss, Wilhelm, & Sander, 2007). The capacity limit of this process has repeatedly been shown to share substantial variance with fluid intelligence (G*f*; e.g., Colom, Abad, Quiroga, Shih, & Flores-Mendoza, 2008; Engle, Tuholski, Laughlin, & Conway, 1999; Fry & Hale, 1996; Kane & Engle, 2002; Kyllonen, 1996; Kyllonen & Christal, 1990; Salthouse, 1992). G*f* is a core component of general intelligence (*g*), and refers to the ability to think logically and solve novel problems (Cattell, 1971). It is considered to be independent of acquired knowledge or cultural influences and has been shown to correlate highly with *g* (Gustafsson, 1984; Snow, Kyllonen, & Mashalek, 1984; Sommer, Arendasy, & Häusler, 2005; Süss, Oberauer, Wittman, Wilhelm, & Schulze, 2002). The close relationship between WMC and G*f* led some researchers to assume that WMC and G*f* are identical constructs (e.g., Colom, Flores-Mendoza, & Rebollo, 2003; Engle, 2002; Kyllonen, 2002; Stauffer, Ree, & Caretta, 1996). Meta-analytical results, however, did not support this idea. Ackerman et al. (2005), for example, reported a latent correlation of *r* = .50 between WMC and G*f* casting some doubt on the assumption that WMC and G*f* are identical constructs. Oberauer, Schulze, Wilhelm and Süss (2005) argued that this is an underestimation because of several methodological shortcomings and biases. These latter authors reanalyzed the data examined by Ackerman et al. (2005) and obtained a correlational relationship of *r* = .85 between the two constructs. Despite the close association between WMC and G*f*, the two constructs were still clearly dissociable from each other (Oberauer et al., 2005).

To date, it is unclear why some results indicate a very strong relationship whilst others reveal only a moderate association between WMC and G*f*. Schweizer (2007) put forward the idea of impurity of WMC measures as a possible explanation of the high correlations found in previous studies. Impurity results from the fact that tasks capturing cognitive functions do not only measure the intended variance due to the process of interest, but also variance caused by other processes, such as basic aspects of information processing. A WMC task, for example, can only be solved when a person is able to perceive the presented stimuli and to pay sufficient attention to the task. Hence, sensory acuity and the participant’s state of alertness may affect performance on a WMC task even though the task intends to measure WMC (and WMC certainly plays a crucial role for task performance). The numerous processes contributing to task performance produce the impurity. Due to impurity the association between G*f* and WMC measures may be overestimated because not only WMC processes but also other processes may have contributed to an observed correlation. If we want to measure pure relationships between specific constructs, we need to decompose the performance stimulated by a measure into different processes and isolate the processes of interest. When the decomposition of the contributing processes is neglected, it remains unclear whether an observed correlation between performance on a particular task (e.g., WMC) and a potentially related construct (e.g., G*f*) is caused by the experimentally induced variance of interest or by an unrelated source of variance (e.g., sensory acuity or general speed of information processing).

As a methodological approach to identify and decompose variance of a given task into independent components and to isolate the processes of interest, Schweizer (2006a, 2006b, 2008, 2009) introduced the so-called fixed-links modeling approach. Fixed-links modeling is a special kind of confirmatory factor analysis (CFA) for data derived from an experimental repeated-measures design. In many WMC tasks, the task demands are systematically increased from easy conditions, with only a small number of items to be stored and processed in working memory, up to highly demanding conditions, with a large number of items. To depict this experimental manipulation of working memory demands, a latent variable can be derived by means of fixed-links modeling with factor loadings fixed in a way that reflects the increasing order of the conditions. Thus, a condition with higher working memory demands gets a higher weight on the latent variable compared to a condition with lower working memory demands. Because the factor loadings are fixed, it is also possible to derive additional latent variables from the same set of manifest variables (i.e., performance measures in the task conditions) as long as the course of the numbers serving as factor loadings differs from each other. If we assume, for example, that variables such as sensory acuity, a person’s general state of alertness, and/or motivation also influence WMC task performance, then the influence of these variables probably varies within, but not among, task conditions in a systematic way. Consequently, a latent variable can be derived from performance measures in the different task conditions with factor loadings fixed to the same value. In case that factor loadings are fixed, the variance of the latent variable is freely estimated and it is necessary that there is a statistically significant amount of variance to indicate that the latent variable reflects a psychologically meaningful process. Thus, while in traditional CFA the variance of the latent variable is fixed to 1 and the factor loadings are freely estimated, in fixed-links models, the factor loadings are fixed and the variance of the latent variable is freely estimated. Furthermore, while in a traditional CFA all common variance of the manifest variables is assigned to one latent variable , more than only one latent variable^1^ can be derived from the same set of manifest variables in a fixed-links model to decompose the influence of different sources of variance.

Recently, Schweizer (2007) used the fixed-links modeling approach to investigate the relationship between individual differences in intelligence and working memory processes. Using the Exchange Test (Schweizer, 1996) as a measure of WMC, Schweizer (2007) identified two independent latent variables. One latent variable represented processes that were independent of experimental manipulation with unstandardized factor loadings fixed to 1. The second identified latent variable represented processes that increased with increasing task demands so that unstandardized factor loadings were fixed in a quadratically increasing way. At this point, it is important to understand that the shape of the course of factor loadings across task conditions depicts the experimental manipulation. Thus, the latent variable represents the processes intended to be measured by the experimental task. For example, quadratically increasing unstandardized loadings over five levels of task demands (e.g., 1, 4, 9, 16, 25) imply that the influence of this process would be the smallest for Condition 1, and 25 times larger for Condition 5. Schweizer (2007) arrived at the conclusion that impurity is a major problem for studies investigating the relationship between G*f* and WMC because after disentangling processes of WMC from processes independent of experimental manipulation the obtained latent relationship between working memory and G*f* was of only moderate magnitude (*r* = .40). In the meanwhile, further studies used fixed-links modeling to analyze processes underlying task performance in various repeated-measures designs (Ren, Schweizer, & Xu, 2013; Schweizer, 2008; Stankov & Schweizer, 2007; Stauffer, Troche, Schweizer, & Rammsayer, 2014; Wagner, Rammsayer, Schweizer, & Troche, 2014). These studies have in common that more processes than only one could be identified to underlie performance measures in the respective cognitive tasks. In addition, the shapes of the courses of factor loadings identified were predominantly linearly or quadratically increasing. This is surprising because experimental repeated-measures designs are usually designed to capture a wide range of ability in a given task. Since in fixed-links modeling (as in traditional CFA) the variance-covariance matrix is used for model estimation, the influence of a process depends on the variance and the covariance of task conditions. In case that conditions of a working memory task exceed the capacity limit of more and more participants, variance of these task conditions should decrease and, similarly, the covariance between difficult task conditions should decrease. Thus, we would expect increasing unstandardized factor loadings from easy conditions to medium-difficult conditions, the highest unstandardized factor loadings for medium-difficult task conditions, and decreasing unstandardized factor loadings for very difficult task conditions. Such a course of factor loadings, however, has not been identified yet, although this shape seems reasonable.

Most importantly for the present study, the latent variables representing processes related and unrelated to experimental manipulation, respectively, were associated differentially to measures of psychometric intelligence in previous studies (Miller, Rammsayer, Schweizer, & Troche, 2010; Ren et al., 2013; Schweizer, 2007; Stauffer et al., 2014; Wagner et al., 2014). The aim of the present study was to systematically compare results obtained by traditional CFA with results provided by the fixed-links modeling approach. Therefore, in this article, we directly contrast the fixed-links modeling approach and traditional CFA by using parts of the data previously reported by Troche and Rammsayer (2009). Using a WMC task consisting of five conditions with increasing demands on WMC, we derived one latent variable from the five task conditions by means of a traditional CFA. Furthermore, by applying the fixed-links modeling approach, we probed whether we could identify more than only one process underlying performance on the WMC task. For both measurement models (traditional CFA and fixed-links model) we investigated the relationship between the derived latent variables and a measure of G*f* derived from subtests of the Berlin Intelligence Structure (BIS) test (Jäger, Süss, & Beauducel, 1997). We acted on the following assumptions:

When applying traditional CFA, a latent variable WMC can be derived from the five conditions of the WMC task, which is closely associated with G*f* as commonly found in research on the relationship between WMC and G*f* (Oberauer, Süss, Wilhelm, & Wittmann, 2008).

Using the fixed-links modeling approach, we assume that two latent variables can be identified to explain variance within and covariance between the five WMC task conditions. One latent variable represents processes involved in WMC task performance but independent of experimental manipulation so that the unstandardized factor loadings can be fixed to 1. The second latent variable represents processes varying with experimental manipulation (i.e., increasing demands on WMC). Consequently, the unstandardized factor loadings systematically vary with experimental manipulation. However, although we expect systematic variation across task conditions, it is difficult to predict the exact course of factor loadings across conditions of a given task. Therefore, we compare different courses for the latent variable representing WMC. In line with previous studies, we probe linearly and quadratically increasing functions to represent WMC (as reported by Schweizer, 2007, 2008; Stauffer et al., 2014). As the applied WMC task put heavy demands on WMC in the most difficult task conditions, it is possible that these excessive task demands result in reduced variance of conditions and, consequently, in reduced covariance between conditions. This should induce a flattening or even a decline of the function so that we also probe a logarithmic as well as a reversed u-shaped function with increasing factor loadings in the first conditions and decreasing factor loadings for the most difficult conditions.

After having identified a latent variable representing a purified measure of WMC, we expect a positive functional relationship between WMC and G*f*. We assume, however, that this correlation is significantly smaller than the correlation obtained by relating the WMC measure from the traditional CFA to G*f*. Even more important, if an additional latent variable can be identified representing processes unrelated to experimental manipulation, this latent variable should be related to G*f*. It is this relationship that should lead to an overestimation of the WMC-G*f* relationship when the underlying processes are not disentangled.

## Method

### Participants

Participants were 100 male and 100 female volunteers ranging in age from 18 to 30 years (*M*_age_ = 22.2, *SD*_age_ = 3.3 years). To cover a large range of individual levels of psychometric intelligence, participants with different educational backgrounds were recruited. Ninety-three participants were university students, 89 participants were vocational school students and apprentices, and 18 participants were working individuals of different professions. All participants were informed about the study protocol and gave their written informed consent.

### Measurement of reasoning

Subtests of the BIS test (Jäger et al., 1997) were administered to obtain a measurement of reasoning. The BIS test is a paper-pencil test based on Jäger’s (1984) BIS model of intelligence. According to the BIS model, cognitive abilities can be classified along two modalities: the content of a given task and the mental operation required to solve the task. Three contents (verbal, numerical, and figural) and four operations (reasoning, speed, memory, and creativity) are differentiated. From tasks with different contents but requiring the same operation (e.g., verbal, figural and numerical reasoning tasks) the respective operation-related intelligence can be inferred (e.g., reasoning). Analogously, from tasks with the same content but requiring different operations (e.g., a verbal speed, a verbal reasoning, a verbal memory and a verbal creativity task) an index for the respective content-related intelligence can be derived (e.g., verbal intelligence). Furthermore, on a more abstract level, Jäger (1984) assumes a general factor of psychometric intelligence as an integral of all human abilities. More details about the BIS model can be found in Jäger et al. (1997) or in Bucik and Neubauer (1996).

Subtests of the BIS test were administered to the participants in small groups of 2 to 5 participants each. For the present study, six reasoning subtests from the BIS test (Jäger et al., 1997) were chosen with two subtests for each content (figural, numerical, verbal). We decided to use reasoning subtests only because of the close association between reasoning and G*f* (Süss et al., 2002) which has been of particular interest in previous research on the relationship between WMC and intelligence. The figural reasoning tests required recognition of figural analogies (Reasoning F1) and completion of a progressing string of figures (Reasoning F2). The numerical reasoning tests comprised continuation of number series (Reasoning N1) and estimation of mathematical solutions (Reasoning N2). In the verbal reasoning tests, semantic relations should be recognized (Reasoning V1) and semantic relations between words should be judged (Reasoning V2). Performance scores of all six subtests were z standardized. Normalized scores of the two verbal, numerical, and figural reasoning subtests were averaged to obtain three reasoning scores (one verbal, one numerical, and one figural score). By using CFA, these three reasoning scores allowed us to build one latent variable Reasoning (see Jäger et al., 1997 for further information).

### WMC task

To measure WMC, a computer-based adaptation of Oberauer’s (1993) figural dot span task was used. This task has been shown to be a valid measure of WMC (e.g., Süss et al., 2002).

#### Apparatus and Stimuli

**Figure 1.**
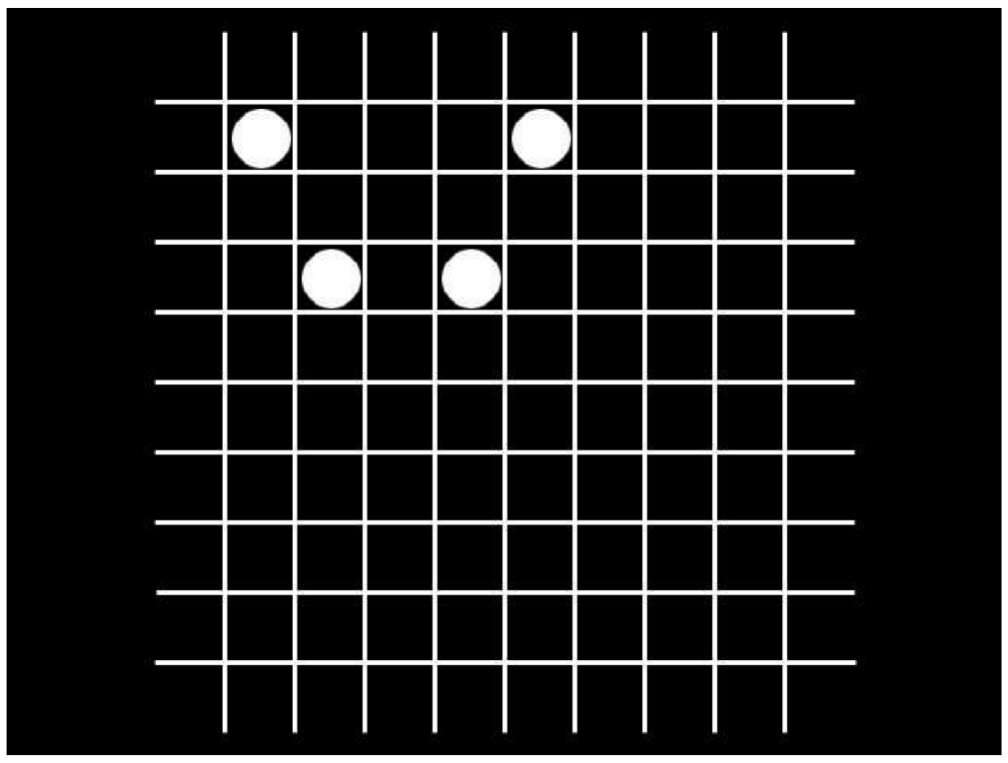
Example for a vertically symmetrical dot pattern of condition three.

Visual stimuli were white dots with a diameter of 2.2 cm presented within a 10 × 10 grid on a 19’ computer monitor (ViewSonic VX924). The grid consisted of white lines against a black background and had a size of 26.5 × 26.5 cm (see Figure 1). Participants’ responses were registered by an optical computer mouse.

#### Procedure

About one week after the intelligence testing session, participants were reinvited for the experimental session in which they were separately tested in a sound attenuated room. The WMC task consisted of five conditions with three trials each resulting in a total of 15 trials. The five conditions differed from each other in the number of dots presented during a trial with two dots in the first and six dots in the fifth condition. A trial started with the presentation of the grid. On each trial, two to six white dots were presented successively in different cells of the grid for one second each. The interstimulus interval (ISI) between the dots was 500 ms. Participants’ task was to memorize the spatial positions of the dots. After presentation of the dots, participants had to answer whether the array of the dots would have been horizontally or vertically symmetrical if all dots were visible concurrently.

Participants sat 40 cm away from the computer monitor, and gave their answers by clicking with the computer mouse in one of two designated response areas presented in the middle of the grid. After that, they were asked to click on the cells in which the dots had been presented. Feedback was provided after each trial. For each participant, the five task conditions were given in the same order so that the amount of information to be stored continually increased. As dependent variable, hit rate of correctly reproduced dot positions in each task condition was determined.

### Statistical Analyses

CFA was computed using Mplus software 7.11 (Muthén & Muthén, 2012). Because data were not normally distributed, we used the Satorra-Bentler robust maximum likelihood estimation method (Satorra & Bentler, 1994). This estimation method is suited for not normally distributed data because it has been shown to be a more robust estimator than simple maximum likelihood estimation (Finney & DiStefano, 2006; Kline, 2011). Model fits were evaluated by means of the chi-square (χ^2^) value. The χ^2^ value indicates the similarity between the covariance matrix implied by the researcher’s model and the sample covariance matrix. A significant χ^2^ value (*p* < .05) denotes that the discrepancy between implied model structure and empirical data cannot be ascribed to sampling error alone and, thus, designates a poor model fit. As in every other test statistic, the statistical power not to reject the alternative hypothesis, although it is true, is a positive function of sample size. That is, the larger the sample size, the higher the probability of accepting the alternative hypothesis. When applying CFA, this poses a problem, because rejecting the null hypothesis (*p* < .05) indicates model misspecification. To circumvent this problem, we additionally used approximate fit indices. In simulation studies, approximate fit indices have been shown to be less affected by sample size than the χ^2^ value (Cheung & Rensvold, 2002; Meade, Johnson, & Braddy, 2008). The following fit indices were applied: Comparative Fit Index (CFI; Bentler, 1990), Root Mean Square Error of Approximation (RMSEA; Steiger, 1989), and the Standardized Root Mean Square Residual (SRMR; Bentler, 1995). Hu and Bentler (1999) regard CFI .95 and SRMR .08 as a good model fit. Also an RMSEA .05 indicates a good model fit (Browne & Cudeck, 1993). For model comparison, we used the Akaike Information Criterion (AIC; Akaike, 1987) and the CFI. When comparing two models, a model is better than the other one when its AIC is lower (indicating higher parsimony) and its CFI is more than .01 larger compared to that of the other model (Cheung & Rensvold, 2002). More detailed information on the applied fit indices is provided by Hu and Bentler, and Schermelleh-Engel, Moosbrugger and Müller (2003).

In a first step, we estimated the relationship between WMC and G*f* using traditional CFA. That is, we derived a first latent variable “G*f*” from the three aggregates of verbal, numerical, and figural BIS-Reasoning subtests and a second latent variable from the hit rates in the five conditions of the WMC task. The obtained regression coefficient between the two latent variables served as a reference value for the coefficients obtained by the fixed-links modeling approach.

In a second step, we decomposed the variance of the WMC task into independent processes represented by two latent variables. The first latent variable was assumed to represent processes that were not affected by increasing task demands and, thus, did not vary between the experimental conditions. Therefore, unstandardized factor loadings for all five task conditions were fixed to 1. The second latent variable was assumed to represent WMC-related processes that varied as a function of task demands and, consequently, factor loadings were fixed according to a systematic variation. More specifically, we tested whether this variation might be best described by the assumption of linear, quadratic or logarithmic increase across task conditions, or even by a reversed u-shaped course with an increase from the easy to the medium-difficult conditions and a decrease from the medium-difficult to the very difficult conditions. After having identified the best measurement model by means of fixed-links modeling, the relationship was computed between G*f* and the latent variables representing processes related and unrelated to the present WMC manipulation, respectively.

## Results

Descriptive statistics of scores on the six BIS subtests and performance measures of the WMC task are given in Table 1. Pearson correlations among intelligence scores and performance measures can be seen from Table 2. An additional analysis yielded good internal consistency for both BIS-Reasoning (α = .79) and the WMC task (α = .82).

**Table 1.** Descriptive Statistics of Scores on the BIS Subtests and Performance Measures of the WMC Task

	BIS-Reasoning Subtests			
	M	SD	Min	Max
Figural 1	3,41	1,59	0	8
Figural 2	2,57	1,71	0	6
Numerical 1	4,03	2,43	0	9
Numerical 2	3,73	2,07	0	7
Verbal 1	3,01	1,91	0	8
Verbal 2	4,96	2,03	0	9
	WMC task			
Condition 1	.88	.18	.17	1,00
Condition 2	.77	.21	.22	1,00
Condition 3	.77	.19	.17	1,00
Condition 4	.61	.23	.13	1,00

*Note. N* = 200.

**Table 2.** Correlations Among Different Measures of Reasoning and Hit Rate on Experimental Conditions of the WMC Task

	BIS-Reasoning		WMC task				
	Numerical	Verbal	Condition 1	Condition 2	Condition 3	Condition 4	Condition 5
BIS-Reasoning							
Figural	.58***	.40***	.36***	.28***	.41***	.43***	.34***
Numerical		.42***	.27***	.28***	.40***	.40***	.32***
Verbal			.17*	.15*	.27***	.21**	.15*
WMC task							
Condition 1				.38***	.52***	.39***	.42***
Condition 2					.56***	.45***	.42***
Condition 3						.55***	.49***
Condition 4							.53***

*Note.N* = 200, *p < .05, **p < .01, ***p < .001 (two-tailed).

### Traditional CFA

**Figure 2.**
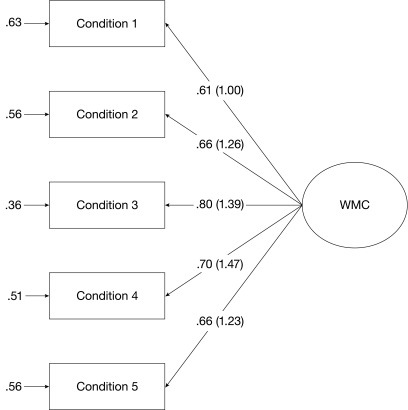
Congeneric model of measurement of WMC with standardized (unstandardized) factor loadings (Model 1).

Significant positive correlations among the five conditions of the WMC task were indicative of a latent variable underlying task performance. Therefore, in a first step, we conducted traditional CFA according to the congeneric measurement model (Jöreskog, 1971) for WMC. The congeneric model of measurement represents the most popular way to describe the empirical data by a single factor. Loadings of the utilized indicators were freely estimated. This Model 1, presented in Figure 2, yielded a good model fit with a non-significant Satorra-Bentler corrected (SB) χ^2^ value and good approximate fit indices (see Table 3).

**Table 3.** Fit Statistics for the Congeneric (Model 1) and the Fixed-Links Models (Models 2 to 5)

	Represented processes	SB ÷2	df	P	CFI	RMSEA	SRMR	AIC
Model 1	Congeneric	7.5	5	.19	.992	.05	.02	-2.5
Model 2	Constant + Linear	14.07	8	.08	.980	.06	.05	-1.93
Model 3	Constant + Quadratic*	15.16	8	.06	.977	.07	.06	-0.84
Model 4	Constant + Logarithmic	12.84	8	.12	.984	.06	.05	-3.16
Model 5	Constant + iu-shaped	8.6	8	.38	.998	.02	.053	-7.4

*Note.Note*. Constant: constant processes, Linear: linearly increasing processes, Quadratic: quadratically increasing processes, Logarithmic: logarithmic processes, iu-shaped: inverted u-shaped processes, SB χ^2^: Satorra-Bentler corrected χ^2^ value, CFI: comparative fit index, RMSEA: root mean square error of approximation, SRMR: standardized root mean square residual, AIC: Akaike information criterion. * The variance of the dynamic latent variable did not reach statistical significance.

**Figure 3.**
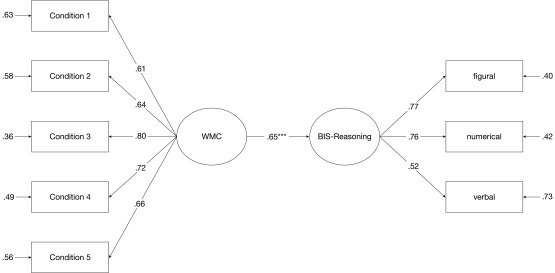
The relationship between BIS -Reasoning and WMC as derived from a traditional CFA. All coefficients are standardized. ***p < .001 (two-tailed).

We derived a latent variable BIS-Reasoning from the three reasoning scores and regressed this latent variable on WMC as measured in Model 1 (see Figure 3). The fit of this model was good with a non-significant SB χ^2^ value and good approximate fit indices [SB χ^2^(19) = 18.82, *p* = .47, CFI = 1.000, RMSEA = .00, SRMR = .03, AIC = -19.18]. Using the traditional CFA for the measurement model of WMC, there was a strong association between WMC and BIS-Reasoning (β = .65, *p* < .001).

### Fixed-links modeling

Next, we used a fixed-links modeling approach to analyze our data. We examined whether two independent latent variables might explain variance and covariance of hit rates in the five WMC task conditions. The first latent variable represented processes not varying with experimental manipulation (e.g., sensory acuity or general speed of information processing). The second latent variable represented processes that vary systematically with task condition (i.e., WMC load). For reasons of brevity, we refer to the latent variable with constant factor loadings across the five task conditions as “constant latent variable” and to the latent variable with factor loadings systematically varying with task conditions as “dynamic latent variable”. In Model 2, the constant latent variable had its unstandardized factor loadings of all five task conditions fixed to 1. The dynamic latent variable, however, had increasing factor loadings across the five task conditions. As task difficulty of the WMC task increased linearly across the five conditions, we tested a linearly increasing function (i.e., .1, .2, .3, .4, .5). The two latent variables were set to be independent of each other. Model 2 had a good model fit with a non significant SB χ^2^ value and good approximate fit indices (see Table 3). The variance was .0157 (*z* = 5.49, *p* < .001) for the constant latent variable and .0353 (*z* = 2.06, *p* < .05) for the dynamic latent variable representing the experimental manipulation of WMC demands.

Because identifying a model with a good fit does not necessarily imply that the model is true (MacCallum & Austin, 2000), we tested alternative models to see if there are models fitting the data even better. More specifically, we tested functions of factor loadings which have been suggested in previous work (cf., Schweizer, 2007). In Model 3, we replaced the linearly increasing function of factor loadings of the dynamic latent variable by factor loadings following a quadratic function (i.e., .01, .04, .09, .16, .25). Model 3, consisting of a constant latent variable and a quadratically increasing latent variable showed a similar model fit to Model 2 with a non-significant SB 2 value and good approximate fit indices (see Table 3). The variance was .0172 (*z* = 6.98, *p* < .001) for the constant latent variable and .1306 (*z* = 1.74, *p* = .08) for the dynamic latent variable representing the experimental manipulation of WMC demands. Because the variance of the dynamic latent variable did not reach statistical significance, this latent variable does not reflect a psychologically meaningful process. Consequently, despite its good model fit, this model was rejected and discarded.

In Model 4, we replaced the unstandardized factor loadings of the dynamic latent variable of Model 2 by factor loadings following a logarithmic function (i.e., .30, .48, .60, .70, .78). The assumption of a logarithmic function was based on the consideration that, in real-life situations, a linearly increasing process appears rather implausible. A linearly increasing function would imply an ever-increasing influence of the latent variable with increasing task demands. From a practical standpoint and in due consideration of the performance data obtained in the present experiment, this seems rather unlikely. In Conditions 4 and 5 of our WMC task, where participants had to memorize five and six dots, respectively, task demands were so high that participants reached their WMC limit as indicated by the hit rate of these experimental conditions (see Table 1). Model 4, consisting of a constant latent variable and a logarithmically increasing dynamic latent variable showed a good fit with a non significant SB 2 value (see Table 3). Model 4 had the lower AIC value than Model 2 indicating a better model fit for the former model. Variances were .0132 (*z* = 3.74, *p* < .001) for the constant latent variable and .0176 (*z* = 2.34, *p* < .05) for the dynamic latent variable.

**Figure 4.**
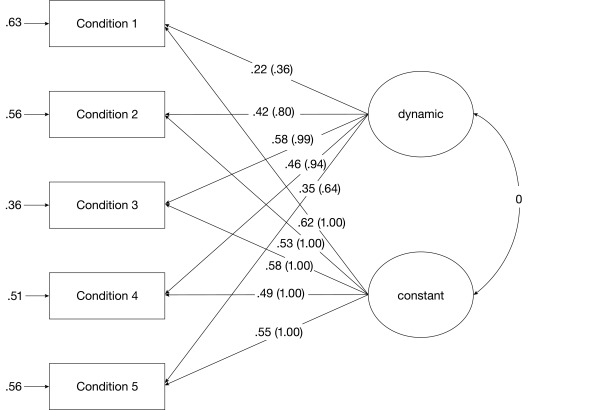
Fixed-links model of measurement of WMC (Model 5) with standardized (unstandardized) factor loadings.

In Model 5, we took the assumption of people reaching their WMC limit a step further. More specifically, we replaced the unstandardized factor loadings of the dynamic latent variable of Model 4 by factor loadings following an inverted u-shaped function (i.e., .36, .80, .99, .94, .64). This series of factor loadings unfolds when the x-values of -8, -4.5, -1, 2.5, and 6 are used with the function *f*(*x*) = (-*x*^2^ + 100)/100. Choosing this function allowed us to use unstandardized factor loadings ranging between 0 and 1 following an inverted u-shaped function. This function implies that participants not only reached their WMC limit but also exceeded it. The excessive demands on processes required to solve the WMC task in the most difficult conditions potentially led to a decrease of variance because more and more individuals failed to solve the task. This effect is described by a decrease of unstandardized factor loadings from the third to the fifth condition. Model 5 is presented in Figure 4 and yielded a good model fit with a non-significant SB χ^2^ value and good approximate fit indices (see Table 3).

The variance was .0120 (*z* = 3.80, *p* < .001) for the latent variable representing constant processes of information processing and .0121 (*z* = 3.17, *p* < .01) for the dynamic latent variable representing experimentally induced WMC-specific processes. Besides the good model fit, Model 5 yielded a better fit and was more parsimonious than Models 1, 2, and 4, as indicated by a higher CFI and a smaller AIC value (see Table 3). To test whether the series of factor loadings of Model 5 was robust, we randomly assigned each participant into one of four groups. Then, in a next step, we tested Model 5 four times leaving out each group once. Neither of the four 2 tests reached statistical significance (all *ps* > .35). Based on these findings, we inferred that the obtained inverted u-shaped function was robust and did not capitalize on chance.

**Figure 5.**
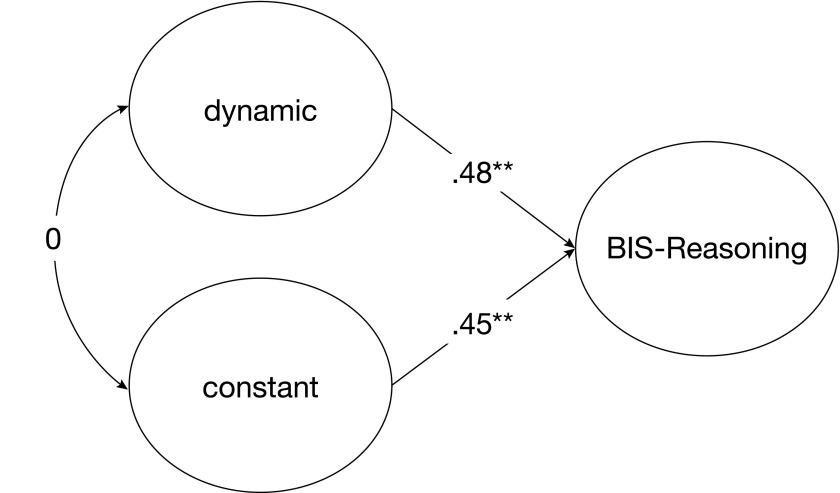
The relationship between BIS-Reasoning and two latent variables derived from the WMC task by means of fixed-links modeling (Model 5). The dynamic latent variable represents WMC-specific processes. Regression coefficients between latent variables are standardized coefficients. **p < .01 (two-tailed)

As Model 5 described the data better compared to Models 2 and 4, we assumed that the WMC-related processes of this task are best represented by the latent variable with factor loadings showing an inverted u-shaped function across task conditions. Proceeding from this assumption, we probed how this measure of WMC, provided by the fixed-links modelling approach, is related to BIS-Reasoning. For this purpose, we regressed the BIS-Reasoning factor derived from the three reasoning scores on the constant and dynamic latent variables of Model 5. The structural part of this model is depicted in Figure 5. Its model fit was good with a non-significant SB χ^2^ value and good approximate fit indices [SB χ^2^(21) = 20.27, *p* = .50, CFI = 1.000, RMSEA = .00, SRMR = .04, AIC = -21.73]. The regression coefficients of BIS-Reasoning on the dynamic as well as the constant latent variable were = .48 (*p* < .01) and = .45 (*p* < .01), respectively.

At first sight, the outcome of the present study suggests that a purified measure of WMC load, as obtained by fixed-links modelling, is less strongly related to BIS-Reasoning (β = .48, *p* < .01) than the impure measure obtained with traditional CFA (β = .65, *p* < .001; see Figure 3). To examine whether these two coefficients were significantly different from each other, we constrained the parameter between BIS-Reasoning and WMC obtained with fixed-links modeling to β = .68, as obtained with traditional CFA. A Wald test indicated no statistically significant difference [χ^2^(1) = 3.00, *p* = .08] between the constrained model and the unconstrained model. This result indicates that fixing the parameter β = .48 to β = .68 does not impair the model fit although WMC, as depicted by traditional CFA, shared 42.25% of variance with Reasoning but only 23.04% when measured by fixed-links modeling.

Most importantly, however, the latent variable representing processes independent of experimental manipulation was also substantially related to BIS-Reasoning (β = .45, *p* < .01) with 20.25% of common variance. Thus, virtually the same portion of variance of approximately 43% in BIS-Reasoning was explained by the two latent variables derived by means of the fixed-links modelling approach, on the one hand, and by the WMC latent variable obtained by traditional CFA, on the other one. However, it is the fixed-links modeling approach which shows that a significant portion of this variance is not explained by the present WMC load manipulation but by processes unrelated to this manipulation.

## Discussion

Significant positive correlations among task conditions usually give rise to the assumption that one latent variable underlies task performance. There are numerous processes, however, contributing to performance not specific to the cognitive function under investigation (e.g., general sensory acuity or encoding processes in a WMC task). This impurity of measures can lead to false conclusions when estimating the relationship between two latent constructs. The fixed-links modeling approach offers a feasible way of decomposing variance in a repeated-measures design into functionally independent components. After decomposing function-specific processes from subsidiary processes, purified measures prevent drawing invalid conclusions from latent relationships. We systematically compared results obtained by traditional CFA with results provided by the fixed-links modeling approach. We assumed that after deriving two latent variables (as opposed to traditional CFA, where only one latent variable would be extracted) out of a WMC task, the relationship between a latent variable representing WMC specific processes and G*f* should be less pronounced compared to the relationship between G*f* and WMC as a latent variable obtained with traditional CFA. Our results partly confirmed our assumption as the fixed-links model described the empirical data better and more parsimoniously than traditional CFA.

The dynamic latent variable reflects the experimental manipulation of an increasing number of dots to be stored and processed in working memory. From this perspective, the dynamic latent variable represents WMC load - that is, the amount of information to be stored and processed (Daneman & Carpenter, 1980). However, also alternative views of WMC, such as the maintenance of arbitrary bindings (cf. Oberauer et al., 2007), can explain the nature of the dynamic latent variable. Previous studies identified quadratically increasing functions to depict WMC demands (e.g., Schweizer, 2007). In the present study, the dynamic latent variable, however, had factor loadings following an inverted u-shaped function. The fact that the factor loadings decreased from the third to the fifth condition might be explained by the increasing level of task difficulty and the corresponding decrease in variance. Based on these considerations, it appears reasonable to assume that, in the present study, at least some levels of task difficulty were higher than those applied in the previous studies. Nevertheless, our analyses confirmed that the results by Schweizer (2007) obtained with the Exchange Test (Schweizer, 1996) can be generalized to other WMC tasks. The exact course of factor loadings across conditions, however, seems to be rather task specific. Despite our focus on WMC-specific processes constituting the core of the dynamic latent variable, this variable is best understood as a bundle of processes systematically varying with task manipulation. For example, a recent study by Van der Lubbe, Bundt and Abrahamse (2014) suggested a strong functional overlap between working memory and spatial attention. Thus, the dynamic latent variable comprises different processes involved in WMC, such as memory load or aspects of executive and spatial attention (Kane & Engle, 2002; Silk, Bellgrove, Wrafter, Mattingley, & Cunnington, 2010). However, these assumed processes cannot be unambiguously disentangled as long as they vary with experimental manipulation. Future work has to address this important issue.

Of particular interest for the present purpose was our finding that a second latent variable, representing processes unrelated to WMC load manipulation, was identified and shared a substantial portion of variance with G*f*. This constant latent variable reflects processes independent of WMC manipulation but related to G*f*. Again, similar to the interpretation of the dynamic latent variable, the constant latent variable comprises a bundle of processes rather than just one particular process. These “constant” processes include, for example, general (i.e., task-independent) speed of information processing (Stauffer et al., 2014) or basal aspects of sensory acuity (Troche, Wagner, Voelke, Roebers, & Rammsayer, 2014). In addition, also a participant’s current mental or physical state, such as subjective alertness or fatigue, or the individual level of motivation to perform, represent task-independent, constant processes. It should be noted though that the constant latent variable and the dynamic latent variable contributed about equally to G*f*. This finding implicates that both these latent variables are of equal importance when predicting G*f*.

In contrast to our initial expectations, however, a purified measure of WMC load manipulation (the dynamic latent variable) obtained by fixed-links modeling did not show a significantly weaker relation to G*f* than an impure measure obtained with traditional CFA (this absence of statistical significance may be attributable to a lack of power). Nevertheless, our results underline that WMC tasks contain variance unrelated to WMC specific functioning but systematically related to G*f*. This might lead to an overestimation of the relationship between WMC specific processes and G*f* when different sources of variance underlying WMC task performance are not disentangled.

Taken together, the present study documented that impurity may cause a major problem when investigating correlates of psychometric intelligence or, more specifically, G*f*. Furthermore, the fixed-links modeling approach proved to be a useful methodological tool in cognitive psychology for a more valid investigation of the functional relationship between specific constructs than traditional CFA.

## Footnotes

1 Exceptions are the bifactor model (Schweizer, Altmeyer, Reiss, & Schreiner, 2010) and multitrait-multimethod models (Eid, Lischetzke, & Nussbeck, 2005) where also more latent variables than only one are extracted from the same set of manifest variables. For these models, however, factor loadings are not fixed in accordance with theoretical expectations, as it is the case with fixed-links modeling.
